# Staged treatment of a comminuted femoral fracture with Masquelet technique and 3D printed reposition guides

**DOI:** 10.1016/j.tcr.2022.100723

**Published:** 2022-10-17

**Authors:** Raymond O Schaefer, Sophie C Eberlein, Frank M Klenke, Johannes D Bastian, Andreas Hecker

**Affiliations:** Department of Orthopaedic Surgery and Traumatology, Inselspital, Bern University Hospital, University of Bern, Switzerland

**Keywords:** Femoral fracture, Malrotation, Malunion, 3D, Comminution, Reposition guides

## Abstract

**Background:**

Comminuted femoral fractures pose a challenge to the trauma surgeon due to the absence of bony references during surgery. Therefore, malalignment of length and axis can occur and necessitate revision surgery. During the last decade, 3D-planning has evolved as a surgical aid in difficult cases.

**Case report:**

An 18-year-old male patient suffered a polytrauma following a motorcycle accident. This report is about the treatment of a 3rd degree open and comminuted fracture of the left distal femur. The fracture was treated with Masquelet's two-staged technique. With the intent of avoiding malalignment, the second stage surgery was performed with the aid of 3D-planned reduction guides. Despite complex fracture pattern, complete fracture union was achieved with acceptable final alignment (side-to-side comparison of length, axis and femoral torsion).

**Conclusion:**

In this case, performing Masquelet's two-staged surgery with the aid of 3D-printed reposition guides yielded favorable results in regards to rotational malalignment. The malrotation of the femur was reduced after the second operation to a clinically acceptable side-to-side difference (10°). This technique remains technically challenging due to soft tissue tension and limited possibility of soft tissue release.

## Background

Comminuted meta- and diaphyseal femoral fractures remain a major challenge for orthopedic trauma surgeons. Establishing correct femoral torsion remains especially challenging [1]. Postoperative rotational errors exceeding 15° are considered a true torsional deformity and occur in up to 40 % of the cases [1]. True torsional deformities are associated with poor clinical outcomes due to persistent disabilities necessitating revision surgery [2], [3]. Moreover, besides malrotation, varus or valgus malalignment also results in altered knee kinematics and can cause early posttraumatic osteoarthritis [4]. The reason for this high malalignment rate is the absence of bony references during surgery [5]. Therefore, the surgeon can only approximate correct rotation, length and axis. For extremely comminuted and intra-articular fractures, open reduction and internal fixation (ORIF) using plate fixation is favored over intramedullary nailing (IMN) [6].

To date clinically relevant malunion following a previous surgery is often treated with corrective osteotomy. Depending on the location of the injury, an intertrochanteric or distal femoral osteotomy can be performed to correct the malalignment [7]. The last decade has seen an increased usage of 3D-planning and 3D-guided surgery. Excellent accuracy has been reported for this technique with postoperative deviations from the plan of <1 mm and 3° in the context of corrective osteotomies [8], [9]. Mostly, external companies perform planning and production of the 3D-guides. The delivery time for the templates usually is more than four weeks. Therefore, it is not possible to use them in acute fracture treatment. Apart from the manufacturing time, additional costs also pose a limiting factor. Costs are dependent on the printing technique type, staff and software [10].

Another aspect of comminuted fractures is relevant bone loss with the need for filling the defect with autograft and/or allograft [11]. For treatment of such bone defects, Masquelet established a two-staged approach that improved fracture healing and reduced non-union rates. The optimal interval between the two stages is four to six weeks [12], [13].

In this case report, we present our treatment approach for an open and comminuted femoral dia-metaphyseal fracture with relevant bone loss. We combined Masquelet's two-staged technique with the surgical treatment supported by 3D-planning and patient-specific 3D-printed surgical reposition guides.

## Case report

**Fig. 1:**
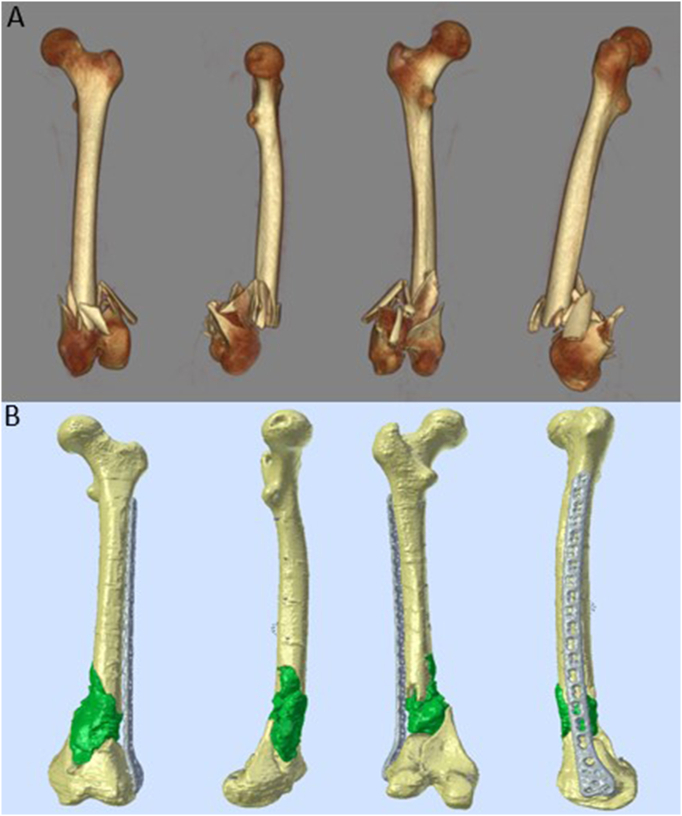
(A) The reconstruction was performed with the hospital's Picture Archiving and Communication System (PACS) after CT scan of the left femur. The distal dia-metaphyseal region has a severely comminuted and displaced fracture with articular extent (AO-classification 33C2.3). (B) 3D-reconstructed model of the femur from CT-data after initial fixation. A lateral condylar bridging plate is in place to stabilize the femur. The green surface indicates the circumferential defect that was filled with bone cement.

An 18-year-old male patient sustained a high-energy trauma following a motorcycle accident. He was transferred to the emergency department of our level one trauma center. Initial whole body computer tomography (CT) revealed multiple fractures (injury severity score = 27), including a 3rd degree open comminuted fracture of the left femur with fracture of the left patella, a 3rd degree open comminuted fracture of the right ankle (open fractures classified according to Gustilo-Anderson), a comminuted left calcaneal fracture, and multiple stable (A0, A1) vertebral fractures. Fig. 1A shows the extent of the femoral fracture.

### Damage control surgery

The patient was always hemodynamically stable and urgently referred to the operating room for damage control surgery. According to international guideline regarding the treatment of open fractures, intravenous antibiotic prophylaxis was initiated preoperatively. Microbiology specimens of the left femur were negative. The open right ankle fracture was contaminated with dirt and biopsy detected pollution with *Pseudomonas fluorescens*. Therefore, the patient received antibiotic therapy. There were no neurovascular damages to the left femur. Debridement and irrigation of the open fractures were followed by external fixation.

### Preliminary internal fixation of the left femur

Six days after admission, preliminary treatment of the left femoral fracture was initiated through a lateral subvastus approach. The external fixation was removed. All fragments in the comminuted area were avascular and therefore removed, leading to a circumferential bone defect of about 10 cm in length. The sagittal split of the condyles that extended into the femoral notch was reduced and held with three 4.5 mm screws leading to a stable joint block. Afterwards an 18-hole variable angle (VA) condylar plate (4.5/5.0 Condylar plate, Synthes, Houston, Texas, USA) was used for internal fixation, following the concept of bridging the defect. Due to the absent bony fracture references, intraoperative determination of length, axis and especially rotation was based on the clinical presentation. The defect was filled with a polymethylmethacrylate (PMMA) cement spacer. Fig. 1B shows the 3D reconstruction of a CT scan of the femur after ORIF and defect filling with bone cement.

### Postoperative 3D-analysis

Alignment analysis based on postoperative CT scans was performed and a consecutive 3D-analysis was conducted by Medacta (MySolution, Medacta International SA, Castel San Pietro, Switzerland). The mirrored contralateral bone was used as a template. A rotational difference of 19° (internal rotation), a coronal axis deviation of 1° (varus) and a flexion malalignment of 3° were detected for the distal femur. Moreover, a shortening of 10 mm, a lateral deviation of 4 mm and an antero-posterior (a.p.) deviation of 8 mm were measured.

**Fig. 2:**
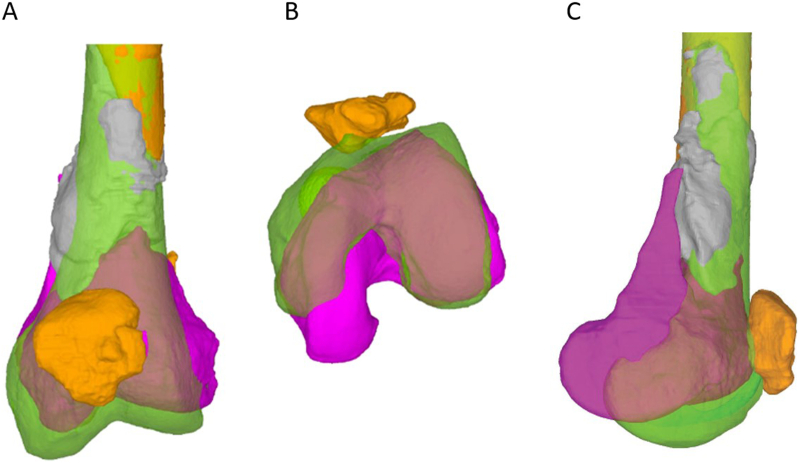
3D-analysis was provided by Medacta (MySolution, Medacta International, Castel San Pietro, Switzerland) after internal fixation. Planning was performed virtually. Analysis of the CT scans of both legs using the mirrored 3D-reconstructed model of the uninjured right femur as reference and a 3D-reconstructed model of the left leg after first stage surgery was performed. To display and measure deviations regarding length, rotation and axis a model of the uninjured right femur (green) was mirrored and superimposed onto the left femur. The left femur is displayed purple distally and orange proximally. Moreover, bone cement (gray) and patella (orange) are shown in coronal (A), axial (B) and lateral (C) view.

The combination of these deviations, but mainly the rotational malalignment led to the decision to revise the alignment during the planned second stage surgery with bone-grafting according to Masquelet. Fig. 2 displays the deviations on a 3D-view.

### 3D surgery plan and creation of 3D-guides

**Fig. 3:**
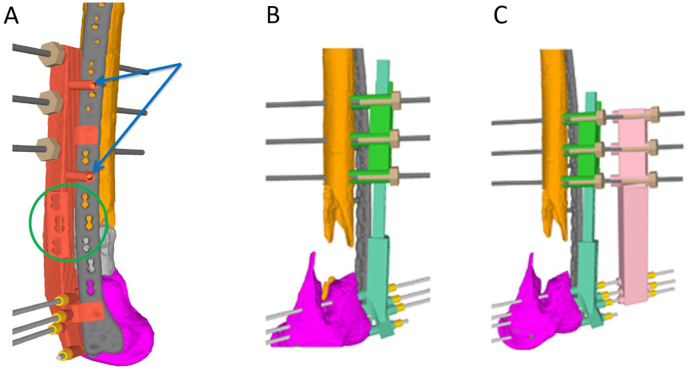
(A) Application of the first guide on the left femur to insert the reference pins. The guide is inserted from anterolateral over the preexisting plate, which provided an excellent reference for the guide. Blue arrows indicate the screw holes in the plate as additional reference for guide placement. The green circle highlights the area for further visual control of the guide position based on the holes in the plate. (B) The reduction guide (green) is slid over the previously set Schanz pins and the length is adjustable using a screw mechanism. (C) A stabilization guide (pink) is placed over the Schanz pins while the first guide is still in place. 3D-analysis was provided by Medacta (MySolution, Medacta International, Castel San Pietro, Switzerland).

The aim of the 3D-planning was to correct the orientation of the distal femoral fragment to the mirrored uninjured side. Subsequently, surgical guides were designed to implement these corrections accordingly. The planning was executed in collaboration between the surgeon and a biomedical engineer. The guides were 3D-printed by Medacta. Surgical planning is displayed in Fig. 3A and B.

### Second stage surgery

Definitive surgical treatment was performed 8 weeks after preliminary internal fixation. Due to the circumferential defect, we decided to add a medial plate via medial subvastus approach to enhance stability [14]. The preexisting lateral approach was used to change the plate. The first guide (Fig. 3A) was placed as planned and the reference pins were inserted. After plate removal, reorientation of the femur was performed using the second set of guides (Fig. 3B). The femoral length was adjusted to the 3D-planning (+10 mm). The reduction maneuver was challenging because the Masquelet-membrane could not be debrided, which led to increased tension when the orientation of the fragments were altered. Angle stable osteosynthesis was performed using a new 18-hole VA condylar plate (4.5/5.0 Condylar plate, Synthes, Houston Texas, USA). The Masquelet-membrane was thoroughly incised longitudinally from the medial side and the cement spacer was removed. Subsequently, the proximal and distal fracture endings were decorticated and multiple 1 mm drilling holes were created to promote blood supply. The defect was filled with autograft from the iliac crest, allograft (femoral head, Smith & Nephew Schweiz AG, Zug, Switzerland) and demineralized bone matrix (GRAFTON® DBM Putty, 5 cc, Medtronic Sofamor Danek Inc.; ratio allograft/autograft 3:1). Afterwards the Masquelet-membrane was closed with absorbable sutures. A 12-hole locking compression plate (4.5/5.0 mm LCP, DePuy Synthes, Houston Texas) was bent in order to achieve a good fit medially and then fixated to the bone with cortical screws. Locking screws were avoided on the medial side, because very stiff constructs can lead to higher non-union rates [13].

### Aftercare

The postoperative care was limited due to multiple injuries on both legs. For the left femur, partial weight-bearing with 15 kg was allowed, but due to the concomitant injuries only wheelchair mobilization was possible. The patient underwent inpatient musculoskeletal rehabilitation for 3 months. Weight-bearing was slowly increased to full weight-bearing at 6 months postoperatively aided by an intensive ambulatory rehabilitation protocol.

### Outcome

**Fig. 4:**
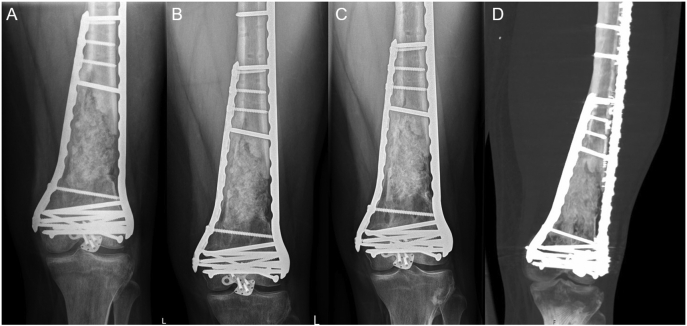
This figure shows the fracture consolidation over time. The radiograph at three months postoperative (A) shows both medial and lateral LCP-plates stabilizing the distal femur and an arrow-shaped plate for osteosynthesis of the patella. There are no signs of loosening or graft resorption. The interposed bone graft is partially integrated. At six months postoperatively (B), progredient consolidation and integration of the interposed bone graft can be stated. 12 months postoperatively, consolidation of the fracture with complete integration of the bone graft and cortical bone formation could be found on the radiographs (C) and computer tomographic imaging (D).

At the final follow-up one year postoperatively, the patient was satisfied with his left hip and knee function and had no pain. The fracture was completely healed confirmed by a CT scan. No signs of infection were present. The patient had an IKDC-score of 67.8 % (International Knee Documentation Committee, score ranges 0–100, where 100 = no limitation with daily or sporting activities and the absence of symptoms). Femoral alignment parameters are displayed in Table 1. Fig. 4 shows the radiographs and a CT scan of the left distal femur.

**Table 1:** 3D-analysis after 1st and 2nd stage surgery.

	After 1st stage surgery	After 2nd stage surgery
Δ femoral torsion	19° (internal rotation)	10° (internal rotation)
Δ length	−10 mm (shortened)	−11 mm (shortened)
Coronal axis	1° (varus)	6° (varus)
Sagittal axis	3° (flexion)	1° (flexion)

The postoperative alignment was compared to the mirrored contralateral femur. Deviations were measured after 1st and 2nd stage surgery based on CT scans after 3D-reconstruction and proximal registration. Angles are given in degree (°) and length in millimeters (mm).

Alignment parameters were obtained from CT scans followed by 3D-reconstruction and registration with the mirrored contralateral proximal femur. The medically certified software Materialise Mimics and 3-matic (Materialise GmbH, Munich, Germany) were used for segmentation, registration and measurement. Determined reduction parameters were rotation, length and coronal/sagittal axis. Parameters are displayed in Table 1.

## Discussion

This case report presents a surgical strategy to treat a severely comminuted fracture of the distal femur with relevant bone defect. A frequent complication of these fractures occurred in our case after initial fracture fixation – a malrotation of almost 20°. This led to the decision to correct the alignment during the second stage surgery which was scheduled in line with the Masquelet-staged surgery. According to current literature, malrotation of >15° will likely cause sequelae like persistent pain, limping or problems with climbing stairs or running [2]. Regarding axis alignment, there is a relative indication for revision if >10° of coronal malalignment is present. In the sagittal plane, indications depend on the functional impairment in hip and knee joint. Length differences of >2 cm might be corrected based on a risk-benefit analysis [15].

In our case, the side-to-side difference in femoral torsion was reduced to 10° and flexional alignment to 1°, which is in the range of normal variations and unlikely to cause any problems [16]. Varus axis however, showed a higher varus deviation of 6° after second stage surgery (1° after first stage). The left femur was still shortened (11 mm), even though a length correction of 10 mm was accounted for during the second stage surgery. The most likely reason for the persisting deviations was the soft tissue tension that made the desired alignment challenging. The priority in this case was to preserve the Masquelet-membrane and blood supply to the fracture zone, therefore excessive debridement that would have been necessary for lengthening was not performed.

Kendoff et al. described a CT-navigated reduction of femoral fractures through noninvasive registration of the contralateral intact femur in three cases (one primary, two secondary). They achieved rotational differences to the uninjured side of <3° [17]. An obvious advantage of their technique is the feasibility in the acute period. Nevertheless, they only included rotation, but no other reduction parameters in their results. However, their technique would have been an alternative in our case. Liodakis et al. presented another reduction technique that included an external reduction guide based on virtual ipsilateral 3D-reduction of every single fragment after external femoral and tibial fixation in one case. Considering severe comminution and/or bone loss, though, virtual 3D-reduction reaches its limits in C-type fractures [18].

To avoid non-union in this case with a relevant bone defect, we utilized Masquelet's technique primarily. A systematic review of 427 cases treated with this technique reported an overall union rate of 89.7 % [19]. Especially in open fractures with a higher risk of infection, this technique has proven to be extremely successful [20], [21]. Secondarily, the 3D-guides were used to attain a more accurate femoral alignment than the free-hand standard. 3D-analysis, surgical planning and guide creation occurred in the eight weeks between the two surgeries.

Given the patient's young age and high demand, we aimed to correct length and malalignment meticulously. Therefore, this effortful patient-specific technique was chosen. This may exceed everyday practice resources and is therefore not recommended for less complex cases.

In this case report, we present the surgical management of a severely comminuted and open femoral fracture. The outcome is favorable, considering the severity of the injury. Therefore, we can recommend the following for similar cases:

- first-stage stabilization with bone cement filling of the defect
- postoperative 3D-analysis of alignment compared to the contralateral bone
- in case of relevant malalignment utilization of patient-specific reduction guides in the context of second-stage surgery.
